# An Analysis of Written and Numeric Scores in End-of-Rotation Forms from Three Residency Programs

**DOI:** 10.5334/pme.41

**Published:** 2023-11-03

**Authors:** Lauren M. Anderson, Kathleen Rowland, Deborah Edberg, Katherine M. Wright, Yoon Soo Park, Ara Tekian

**Affiliations:** 1 Department of Family and Preventive Medicine, Rush University, Chicago, Illinois, US; 2 Department of Family & Community Medicine, Northwestern University Feinberg School of Medicine, Chicago, Illinois, US; 3 Department of Medical Education, University of Illinois Chicago, Chicago, Illinois, US

## Abstract

**Introduction:**

End-of-Rotation Forms (EORFs) assess resident progress in graduate medical education and are a major component of Clinical Competency Committee (CCC) discussion. Single-institution studies suggest EORFs can detect deficiencies, but both grades and comments skew positive. In this study, we sought to determine whether the EORFs from three programs, including multiple specialties and institutions, produced useful information for residents, program directors, and CCCs.

**Methods:**

Evaluations from three programs were included (Program 1, Institution A, Internal Medicine: n = 38; Program 2, Institution A, Anesthesia: n = 9; Program 3, Institution B, Anesthesia: n = 11). Two independent researchers coded each written comment for relevance (specificity and actionability) and orientation (praise or critical) using a standardized rubric. Numeric scores were analyzed using descriptive statistics.

**Results:**

4869 evaluations were collected from the programs. Of the 77,434 discrete numeric scores, 691 (0.89%) were considered “below expected level.” 71.2% (2683/3767) of the total written comments were scored as irrelevant, while 3217 (85.4%) of total comments were scored positive and 550 (14.6%) were critical. When combined, 63.2% (n = 2379) of comments were scored positive and irrelevant while 6.5% (n = 246) were scored critical and relevant.

**Discussion:**

<1% of comments indicated below average performance; >70% of comments scored irrelevant. Critical, relevant comments were least frequently observed, consistent across all 3 programs. The low rate of constructive feedback and the high rate of irrelevant comments are inadequate for a CCC to make informed decisions. The consistency of these findings across programs, specialties, and institutions suggests both local and systemic changes should be considered.

## Introduction

Faculty assessment of resident performance during graduate medical education (GME) clinical rotations is expected as part of the supervisory role and remains a “pillar of any programmatic assessment system” (pg. 20) [1]. The Accreditation Council on Graduate Medical Education (ACMGE) requires continuous monitoring and assessment of resident performance for both formative (for learning) and summative (of learning) purposes. Performance must be documented, and resident data provided to the Clinical Competency Committee (CCC) [2] as well as to the Accreditation Data System. The CCC and program directors use information from these assessments to identify residents’ clinical and academic trajectories using the ACGME milestones [3,4] Easily accessible assessment data are also key for residents to access as part of their own learning and feedback to improve their own performance.

Assessment data from End of Rotation Forms (EORFs) are a major component in both CCC discussions and feedback to trainees [3,5,6,7]. EORFs also form the main basis for promotion and entrustment decisions of learners which programs and faculty rely. Studies evaluating the quality and effectiveness of EORFs have shown EORFs can identify struggling residents in some cases [1,5,8,9,10,11]. A 2016 study found EORF scores between problem residents, as identified by the CCC, and non-problem residents were significantly different across all Milestones [8]. Another study found that an EORF can identify problem residents by comparing the comments and scores of those who were put on academic warning or probation and those who were not [9].

Despite the wide prevalence of EORFs and their need in GME, previous studies have found EORFs are flawed at evaluating comprehensive resident performance. When providing numeric ratings, evaluators systematically fail to use the lower end of the scale, creating rating errors [12,13,14,15]. Rating errors such as range restriction (failure to use full scale), leniency or severity error (distributional errors, “*hawks and doves*”), correlational error (give similar ratings across regardless of actual performance) and halo error (inflation) are all commonly found in assessment [16]. A 2004 study evaluating a single surgery residency found 18% of residents in need of some kind of remediation never received a score of “good,” “fair,” or “poor” on their EORFs, receiving only scores of “very good” or “excellent” [10]. Like numeric scores, written feedback also has been found to be flawed. Written comments found on EORFs tend to be low-quality, meaning comments are irrelevant or lack specific examples and recommendations for improvement [9,17,18,19].

It is expected that data from assessments can be used by residents to inform their own improvement, outside of the CCC deliberations. A study by Patel (2015) gathered faculty and resident perspectives on the use of EORFs found that faculty both believed that this form of assessment could be “*critical in moving residents forward in their learning*” but also the EORF is a tool to guide residents in their development. While faculty acknowledge the dual uses of these tools the residents feel that that they receive the form too late and therefore has little impact [20]. Another study found that raters took an average of 89 days to complete an EORF and scores with greater delays led to decreased score variability [8].

Despite their flaws, EORFs continue to be widely used [1,12,21,22]. The ACGME acknowledges the use of faculty assessment forms such as EORFs are “routine and necessary in virtually all GME programs” [1] (pg.18). In response to previously noted problems with EORFs, the ACGME recommends focusing “time and energy into faculty development rather than creation of ‘new and better forms” [1] (pg.17). The ACGME requires EORF data [2] or similar rotation summary assessment data, although it also recommends against an “over-reliance” on it. Moreover, EORFs tend to use assessment items and language stemming from competency-based frameworks (e.g., ACGME Milestones) across different institutions, prompting a need for more comprehensive multisite studies, exploring similarities or differences in the use and inferences generated from EORFs at different institutions. A similar need is motivated also for the use of EORFs across specialties.

This study integrates these needs stemming from the field to better understand EORFs across multiple programs, in multiple specialties, and in multiple institutions, exploring their use in summative and formative assessment. By evaluating data from several programs in a cross-sectional manner, we intended to examine the overall landscape of EORF use in programs to attempt to determine whether the problems with EORFs were program-specific or more likely to reflect systemic issues. We sought to describe whether recent changes in recommendations for increased faculty development and changes in use of EORFs- whether adopted or not- were resulting in data likely to be useful for CCCs.

We build on previous work that focused on one program at one institution [11,17,19,23,24,25,26] or studies solely focused on the written comments component [6,27] by examining multiple specialties and types of institutions to explore whether the EORF is an effective method of assessing resident performance, given its prevalence in GME assessment.

## Methods

### Study setting and participants

We examined assessment data from three residency programs. Program 1 represents an internal medicine (IM) program (resident n = 38; faculty n = 184) from a public, safety-net, urban hospital. Program 2 represents an anesthesia program (resident n = 9; faculty n = 58) from the same public hospital. Program 3 represents an anesthesia program (resident n = 11; faculty n = 57) at large academic suburban medical center. Faculty from each program included both evaluators from inside and outside the department that housed the program. External faculty from Program 3 were not uniquely identified.

Data were collected retrospectively. All End-of-Rotation Forms for a single class from the beginning of residency through graduation were included in the analysis. Our inclusion criteria included residents who completed the program. At Program 3, a four-year program, three residents started the program in year two and were included in the analysis.

### Data collection

All EORFs (numeric scores and written comments) completed by faculty from each program were extracted from the respective residency management systems. Only assessments classified as an EORF were included in the study; non-EORFs, such as an SCO (structured clinical observation) or a similar “on-demand” work-based assessment (WBA), were excluded. Peer evaluations and EORFs completed by evaluators other than faculty were excluded from this study. We redacted identifying information, including names or other information found in comments, prior to analysis. A total of 4,869 forms were collected from the three programs.

### Faculty development

Two of the programs conducted training on how to assess residents; one offering quarterly faculty development sessions focused on evaluation and feedback. No program offered any development on writing comments.

### Data analysis: *Numeric scores*

Program 1 used a three-point rating scale of “below level,” “at level,” and “above level” with additional option for “not observed” for all the EORFs, the number of numeric items ranged from 10–23 (plus comment boxes) depending on the rotation, Similarly, Program 3 used the same 3-point rating scale with a “N/A” option. The forms ranged in length from 5–17 numeric questions plus comment boxes. Program 2 used a yes, no or “not observed” scale; the number of numeric questions ranged from 14–39 items plus comments.

Descriptive statistics were generated using IBM SPSS Statistics for Windows, Version 26 (Armonk, NY: IBM Corp) for all numeric responses. Data were summarized for each year of training and rotation.

### Data analysis: *Written comments*

Written comments were extracted as part of the EORF and separated from the numeric scores. Both comments connected to individual questions and overall comments were included in the analysis. A total of 3,767 written comments were collected from the three programs.

Comments were analyzed for relevance and orientation using a previously developed quantitative coding rubric for the nature of feedback in narrative comments [23,24]. Using the coding rubric as a guide, each comment was assigned two scores, one for relevance and one for orientation using a 4-point coding schema.

A highly relevant (rated 4) comment included specific items that could be used to improve or sustain practice, help the CCC to make competence decisions, or help implement a learning plan. A relevant (rated 3) comment was helpful but lacked specifics or action. Irrelevant (rated 2) and highly irrelevant (rated 1) comments lacked specific details or often were a list of adjectives (smart, confident, hard worker, and so forth) without context.

Orientation scores reflect whether a comment was praise-oriented or critical/growth needed. Mixed orientation comments (both positive and critical language) were discussed between researchers and placed into one category. A comment was judged positive (rated 3 or 4) if the language was encouraging, complimentary, or identified a behavior to reinforce or continue, with more positive language being adjudicated as high praise. Critical comments (rated 2) were those with negative language or those that identified a behavior to change, stop, or where growth was necessary. More severe language or serious concerns were judged as very critical (rated 1). Additional descriptions are provided in supplemental material.

A sample of thirty comments were independently scored by two researchers for inter-rater calibration of the rubric. The results of the initial sample were then discussed and a consensus for each score reached. Both researchers independently scored the remaining comments for all three programs. All inconsistencies were discussed until agreement reached. The four-point scale was collapsed into the binary categories of “critical or positive” and “relevant or irrelevant” for some of the analysis.

## Results

### Numeric score distribution

**Summary**. A total of 4869 evaluations were collected from the three programs, yielding 77,434 total discrete numeric scores and 3767 total comments. Of the 77,434 total numeric data points, 691 items (0.89%) were scored as “below expectations/expected level” or “no.” (Table 1)

**Table 1:** Distribution of numeric scores from end of rotation forms by program.


		TOTAL EVALS	TOTAL NUMERIC DATA POINTS	BELOW EXPECTED LEVEL	AT EXPECTED LEVEL	ABOVE EXPECTED LEVEL	DID NOT OBSERVE/N/A*

**Program 1**	**Total**	**1855**	**33,569**	**39 (0.1%)**	**23,374 (69.6%)**	**10,156 (30.3%)**	**637**

	Year 1	523	10,149	8 (0.1%)	7280 (71.7%)	2861 (28.2%)	61

	Year2	670	11,730	17 (0.1%)	7923 (67.5%)	3790 (32.4%)	184

	Year 3	662	11,690	14 (0.1%)	8171 (69.9%)	3505 (30%)	392

		**TOTAL EVALS**	**TOTAL NUMERIC DATA POINTS**	**NO**	**YES**		DID NOT OBSERVE/N/A*

**Program 2**	**Total**	**732**	**20,579**	**201 (1%)**	**20,378 (99%)**		**1,938**

	Year 1	141	4252	41 (0.2%)	4211 (99.8%)		401

	Year 2	336	10,190	138 (1.4%)	10,052 (98.6%)		1,068

	Year 3	161	3799	12 (0.3%)	3787 (99.7%)		294

	Year 4	94	2338	10 (0.4%)	2328 (99.6%)		175

		**TOTAL EVALS**	**TOTAL NUMERIC DATA POINTS**	**BELOW EXPECTED LEVEL**	**AT EXPECTED LEVEL**	**ABOVE EXPECTED LEVEL**	DID NOT OBSERVE*

**Program 3**	**Total**	**2282**	**23,286**	**451 (1.9%)**	**17,546 (75.3%)**	**5289 (22.6%)**	**632**

	Year 1	110	1290	34 (2.6%)	841 (65.2%)	415 (32.2%)	5

	Year 2	730	8712	123 (1.4%)	6992 (80.3%)	1597 (18.3%)	280

	Year 3	758	6731	102 (1.5%)	5199 (77.2%)	1430 (21.3%)	259

	Year 4	684	6553	192 (2.9%)	4514 (68.9%)	1847 (28.2%)	88


*N/A/Did not observe are not included in the totals.

**Program 1**. 184 faculty completed 1855 evaluations on 38 residents, resulting in 33,569 individual numeric data points over the three-year IM residency. Of these, only 39 (0.1%) were “below expected” level.

**Program 2**. The faculty evaluation scale used was “yes/no” or N/A for each question. The 732 faculty evaluations collected 20,579 data points over the 4-year training period. Faculty gave 201 “no” scores (1%).

**Program 3**. The program completed 2282 evaluations over the training program from faculty members, generating 23,286 individual data points. 1.9% of total scores (n = 451) were below expected level while 75.3% (n = 17,546) were at expected level.

### Written comment relevance and orientation

**Summary**. In total, 4869 evaluations produced 3767 comments from the three programs. Overall, 28.8% (n = 1084) of the total comments were adjugated as relevant, while 71.2% (n = 2683) of the total comments were scored as irrelevant. 3217 (85.4%) of total comments were scored positive and 550 (14.6%) were judged critical.

**Program 1**. Faculty generated 2306 total comments, of which 654 (28.4%) were relevant, whereas 1652 (71.6%) of comments were deemed irrelevant. Of the 2306 comments, 2127 (92.2%), were positive and 179 (7.8%) were critical, as denoted in Table 2.

**Table 2:** End of rotation form written comment orientation and relevance by program.


		TOTAL EVALS	TOTAL COMMENTS	% OF RELEVANT COMMENTS (NUMBER)	% OF IRRELEVANT COMMENTS (NUMBER)	% OF POSITIVE COMMENTS (NUMBER)	% OF CRITICAL COMMENTS (NUMBER)

**Program 1**	**Total**	**1855**	**2306**	**28.4% (654)**	**71.6% (1652)**	**92.2% (2127)**	**7.8% (179)**

	Year 1	523	775	26.6% (206)	73.4% (569)	93.2% (722)	6.8% (53)

	Year 2	670	843	32.9% (277)	67.1% (566)	91% (767)	9% (76)

	Year 3	662	688	24.9% (517)	75.1% (171)	92.7% (638)	7.3% (50)

		**TOTAL EVALS**	**TOTAL COMMENTS**	**% OF RELEVANT COMMENTS (NUMBER)**	**% OF IRRELEVANT COMMENTS (NUMBER)**	**% OF POSITIVE COMMENTS (NUMBER)**	**% OF CRITICAL COMMENTS (NUMBER)**

**Program 2**	**Total**	**732**	**558**	**25.8% (144)**	**74.2% (414)**	**78.9% (440)**	**21.1% (118)**

	Year 1	141	126	23.8% (30)	76.2% (96)	79.4% (100)	20.6% (26)

	Year 2	336	173	20.8% (36)	79.2% (137)	84.4% (146)	15.6% (27)

	Year 3	161	129	27.9% (36)	72.1% (93)	81.4% (105)	18.6% (24)

	Year 4	94	130	32.3% (42)	67.7% (88)	68.5% (89)	31.5% (41)

		**TOTAL EVALS**	**TOTAL COMMENTS**	**% OF RELEVANT COMMENTS (NUMBER)**	**% OF IRRELEVANT COMMENTS (NUMBER)**	**% OF POSITIVE COMMENTS (NUMBER)**	**% OF CRITICAL COMMENTS (NUMBER)**

**Program 3**	**Total**	**2282**	**903**	**31.7% (286)**	**68.3% (617)**	**72% (650)**	**28% (253)**

	Year 1	110	49	14.3% (7)	85.7% (42)	85.7% (42)	14.3% (7)

	Year 2	730	289	37% (107)	63% (182)	73% (211)	27% (78)

	Year 3	758	341	29% (99)	70% (242)	72.4% (247)	27.6% (94)

	Year 4	684	224	32.6% (73)	67.4% (151)	67% (150)	33% (74)


Percentage of relevant comments include both comments scored as “relevant” and “highly relevant”; Percentage of irrelevant comments includes both comments scores as “irrelevant” or “highly irrelevant”; Percentage of positive comments includes comments scored as “high praise” and “moderate praise”; Percentage of critical comments includes comments scored as “critical” or “very critical”.

**Program 2**. Of the 558 comments generated over the four-year training program, 144 (25.8%) were relevant. 440 comments (78.9%) were positive, and 118 (21.1%) comments were critical.

**Program 3**. Of the 903 faculty written comments, 31.7% (n = 286) of comments were relevant, and 72% (n = 650) were positive, with 28% (n = 253) having a critical orientation.

### Combining orientation and relevance

Comment orientation and relevance were collapsed to binary categories, leaving 4 categories for results: critical/relevant; critical/irrelevant; positive/relevant; positive/irrelevant.

In total, 3767 comments were produced from the three programs. Overall, 63.2% (n = 2379) of the comments were positive and irrelevant while 6.5% (n = 246) were critical and relevant.

**Program 1**. Of 2306 written comments, 1530 (66.4%) were positive but irrelevant while 597 (25.8%) were positive and relevant. A total of 179 (7.8%) comments were deemed critical, of which 57 (2.5%) were critical and relevant as delineated in Table 3.

**Table 3:** End of rotation form written comment combined orientation and relevance by program.


	CRITICAL & RELEVANT	CRITICAL & IRRELEVANT	POSITIVE & RELEVANT	POSITIVE & IRRELEVANT

Program 1 N = 2306	2.5% n = 57	5.3% n = 122	25.8% n = 597	66.4% n = 1530

Program 2 N = 558	7.7% n = 43	13.4% n = 75	18.1% n = 101	60.8% n = 339

Program 3 N = 903	16.1% n = 146	11.9% n = 107	15.5% n = 140	56.5% n = 510

Combined Data N = 3767	6.5% n = 246	8.1% n = 304	22.2% n = 838	63.2% n = 2379


**Program 2**. Of 558 total comments, 339 (60.8%) were positive and irrelevant. 144 comments were scored as relevant (25.8%), of which 101 (18.1%) were positive and relevant and 43 (7.7%) were critical and relevant.

**Program 3**. Of 903 total comments, 510 (56.5%) were positive and irrelevant while 140 (15.5%) were positive and relevant and 146 (16.1%) were critical and relevant.

## Discussion

The results of this study indicate the information obtained from End-of-Rotation Forms is not adequate for providing acceptable, constructive feedback to residents, program directors, or program CCCs. Of the 77,434 discrete numeric scores less than 1% were considered “below expected level” while 63.2% written comments were irrelevant and positive. Just 6.5% of written comments were critical and relevant.

Refraining from using negative ratings has previously been described as “the path of least resistance [12].^”^ The results of this study suggest evaluators do not use the lower part of the qualitative scale when providing summative feedback. This study finds this problem is systemic, affecting 3 programs across all years of training, 2 specialties, and 2 institutions assessed in this study. Earlier studies have identified additional barriers including inadequate faculty development, guilt, concern for the resident’s future, and institutional culture [28].

While residents value written comments [27] they are often not useful or actionable [17,18]. The majority of comments in this study were adjudicated as irrelevant (85%). This was true for both critical (55% of total critical comments) and positive (74% of total positive comments). This high proportion of irrelevant comments makes it harder for residents, program directors, and CCCs to find the relevant comments, and it further risks a reduction in the perceived validity of relevant comments. This study identified these assessment limitations of EORFs which should warrant further developments to the assessment methodology, given their wide use in GME.

Our study supports previous studies that found lack of negative data [10,29] and adds additional information about relevance. Similar to findings by both Tekian (2019) and Raaum (2019) our study found that critical comments were more likely to be judged as relevant compared with positive comments, making them even more valuable for improving performance. However, there were far fewer of them overall, highlighting the need for further faculty development and further development of secure learning and teaching environments in GME.

Previous studies have enumerated flaws with EORFs, leading more recently towards more “on-demand” structured clinical observations (SCOs) which rely on specific and direct observations of learners with immediate feedback rather than at the end of the rotation. A recent study compared the effectiveness of written comments in a traditional EORF and a procedural performance assessment completed immediately after a case. They found that 58.3% of the procedural assessment comments and 10.7% of the EORF comments were considered effective [21]. Similar findings have been shown with written comments and overall expectations ratings when comparing EORFs to P-SCO [29]. Other studies have shown on-demand forms are similar to end-of-rotation forms in producing actionable feedback [30].

When taken with the results of this study, this suggests that one of the biggest gaps may be between the medical education specialists and the GME teachers and trainers. That is, a body of literature indicates the strength of SCO assessment methods. However, with continued barriers to implementation and pressure to produce an evaluation at the end of each rotation, the residents in this study continued to receive EORFs.

### Consequences

The CCC has clear expectations that assessment data will be useful [31], and CCC members report that written comments are important to identify potential concerns [5]. Without robust and accurate EORFs, the CCC does not have the information it needs to make appropriate recommendations about milestone progress and promotion. The low relevance of comments in this study makes written comments unlikely to be sufficient to help a CCC, a program director, or a resident use the comments to improve performance, identify strengths, or assess progress through milestones, especially if they must wade through a body of irrelevant comments to find them. Allowing a resident to continue training without identifying areas of concern can ultimately result in harm to patients, especially if a resident is entrusted with a level of care for which they are not clinically prepared [32,33].

### What’s next for assessment

As noted, solutions may initially focus on local-level changes, including additional formal faculty development. Many programs may require development at every level to effectively implement on-demand or SCO assessments alongside EORFs. Because they receive so little of it, residents may not have skills to manage constructive feedback, and may require coaching to contextualize a score of “below expectations” or a critically oriented comment as an opportunity to improve their performance as a physician.

Focusing on local faculty development alone may be most effective if the underlying problem is limited to individual programs. However, because our study includes programs from different specialties, different institutions, and different types of institutions (publicly funded/safety net and academic medical center funded), our findings suggest a systemic rather than localized problem. Therefore, enduring solutions may additionally require higher level changes, such as training of program directors or DIOs, as well as additional support and input from the ACGME or sponsoring institutions.

Ultimately, effective rotation forms may require graduate medical education to embrace honest, transparent, and constructive feedback. Previous literature has noted the challenges associated with creating a learning environment conducive to constructive feedback [27,34].

### Strengths

This study includes programs from different specialties, different institutions, and different types of institutions (public and academic). This allows us to draw conclusions about more than just a single program. Likewise, the inclusion of 3 programs offers a large dataset, with more than 4800 total evaluations and 3700 written comments available for analysis. This data includes residents over the full cycle of their training, either 3 or 4 years, depending on the residency length.

### Limitations

This study was not designed to allow for program-level or resident-level inferences about the residents’ outcomes. We do not know what proportion of residents required remediation for individual rotations or competencies. Although we are aware of residents leaving a program without completing training, we do not know the reasons for this. For these reasons, future studies with more robust contextual data, as well as studies designed to isolate evaluator and program effects may inform or improve generalizability. We did not examine individual resident outcomes, such as milestone achievement or progression, in response to EORF or other information. Studies in the future may incorporate methodologies that consider cross-classification of data (different subsets of faculty rating different learners), an approach more nuanced than traditional clustering or hierarchical techniques, thereby allowing for the unbalanced nature of the data to be better reflected in the inferences. In the current design, we felt that preserving the data structure in its independent form provides a conservative overview of EORFs, for which clustering and other data adjustment techniques would further refine through more robust estimation of trends.

Our study examined three programs. Although results are generally consistent, we did find differences among these programs. A larger study might allow even better characterization of the problems described here. Where data were missing or where faculty chose “N/A” or “unable to answer,” the data were not included in the analysis. We did not perform any analyses to assess the implications of not including these data in our analysis.

### Future research

The goal of GME is to train residents who are competent to practice independently. Assessments, including EORFs, are a significant component of determining whether residents achieve this standard of independence. Future research in this area may include an examination of whether a certain type of evaluator (e.g., core faculty vs non-core faculty; intra-departmental vs extra-departmental) affects the likelihood of a useful evaluation, as well as an analysis of whether certain competencies (i.e., patient care or systems-based practice) are associated with particular numeric scores or comment orientation or relevance. Studying the contributions of different forms and evaluators could lead to better understanding the value in continuing to use rotation evaluations. Finally, future research may help understand other assessment methods, such as frequent, short observation forms that more accurately and honestly describe resident performance.

## Additional File

The additional file for this article can be found as follows:

10.5334/pme.41.s1 Supplemental Material. Relevance and orientation coding scheme for EORF comments using the coding for nature of feedback in narrative comments rubric.

## Previous presentations

This study has not previously been presented in a peer reviewed setting.
