# Supplementary material for: An Analysis of Written and Numeric Scores in End-of-Rotation Forms from Three Residency Programs
Source: Perspect Med Educ. 2023 Nov 3;12(1):497–506. doi: 10.5334/pme.41 (PMC10624145; doi:10.5334/pme.41)
Supplement: Supplemental Material. — Relevance and orientation coding scheme for EORF comments using the coding for nature of feedback in narrative comments rubric. [file pme-12-1-41-s1.pdf]

Supplemental Material

Relevance and orientation coding scheme for EORF comments using the coding for nature of feedback in narrative comments rubric

Relevance Anchor	Sample Comment	Orientation Anchor	Sample Comment
**Highly Relevant (4)**	*"This was a procedure service rotation- knew indications and contradictions to the procedures we did and explained the same to the patients in a clear and simple way- confident and was able to do the procedures available after one or two demonstrations- unfortunately did not perform any LPs and central lines during the 2 weeks."*	**High Praise (4)**	*"continues to exceed my expectations. X is practicing at the level of a junior faculty member. X will have a bright future as an X."*
**Relevant (3)**	*"was a pleasure to work with, [he/she] was well read on the cases and general anesthesia physiology and pharmacology more than the average CA1. X has excellent bedside manners and professionalism."*	**Moderate Praise (3)**	*"Complete consult notes, well thought out plans, concise presentations."*
**Irrelevant (2)**	*"Helps interns and creates supportive atmosphere."*	**Critical (2)**	*"Please work on completing progress notes in a timely manner and also focus on updating A/P and revising progress note daily."*
**Highly Irrelevant (1)**	*"Such a pleasure to work with."*	**Very Critical (1)**	*"is behind peers in knowledge and skills. At times, it appears [he/she] is disinterested in performing or discussing X. requires a great deal of supervision. can also be unreliable in the OR. (place an IV, start an infusion), does not always complete it (or at least tell me [he/she] was unable to complete the task)."*
